# Supplementary material for: Lactotransferrin Downregulation Drives the Metastatic Progression in Clear Cell Renal Cell Carcinoma
Source: Cancers (Basel). 2020 Mar 31;12(4):847. doi: 10.3390/cancers12040847 (PMC7226440; doi:10.3390/cancers12040847)
Supplement: Supplementary file 1 [file cancers-12-00847-s001.pdf]

Supplementary Information

Lactotransferrin downregulation drives the metastatic progression in clear cell renal cell carcinoma

I-Jen Chiu, Yung-Ho Hsu, Jeng-Shou Chang, Jou-Chun Yang, Hui-Wen Chiu, and Yuan-Feng Lin

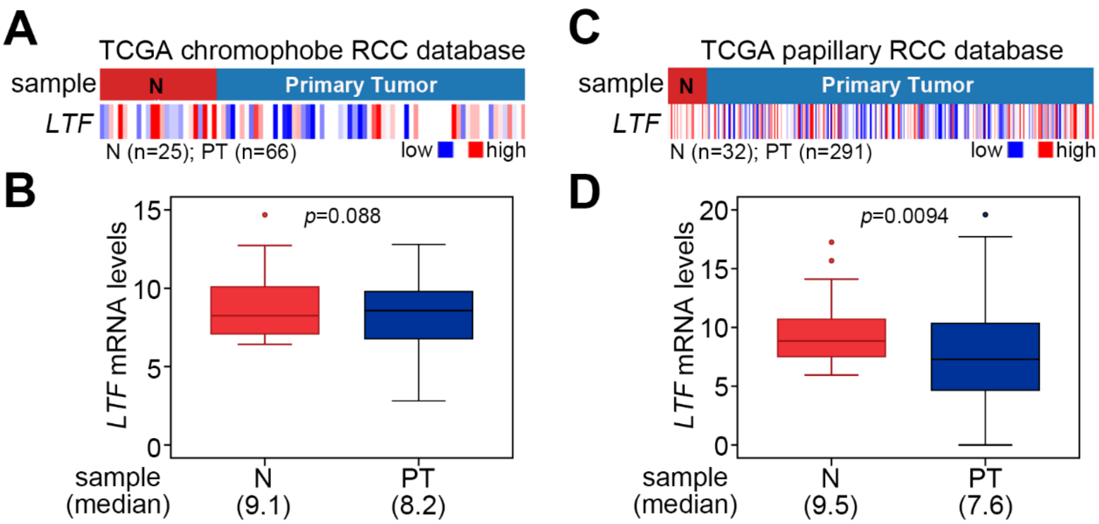

Figure S1. LTF mRNA levels are decreased in primary tumors compared to normal tissues in chromophobe and papillary RCCs. (A and B) Heatmap (A) and boxplot (B) represent the transcriptional profile of the LTF gene in normal tissues (N) and primary tumors (PT) derived from TCGA chromophobe RCC patients. (C and D) Heatmap (C) and boxplot (D) display the transcriptional profile of the LTF gene in normal tissues (N) and primary tumors (PT) derived from TCGA papillary cell RCC patients. In B and D, statistical significance was analyzed by t-test.

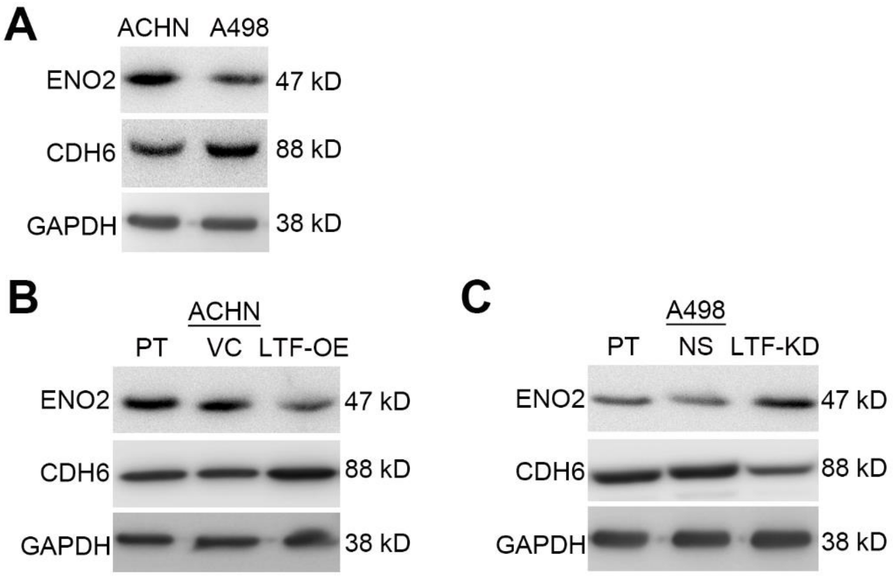

Figure S2. Western blot analyses of ENO2, CDH6 and GAPDH expression in ACHN and A498 cells (A), ACHN cells without (PT) or with VC and LTF-OE (B), or A498 cells without (PT) or with NS and LTF-KD (C). GAPDH was used as an internal control of protein loading.

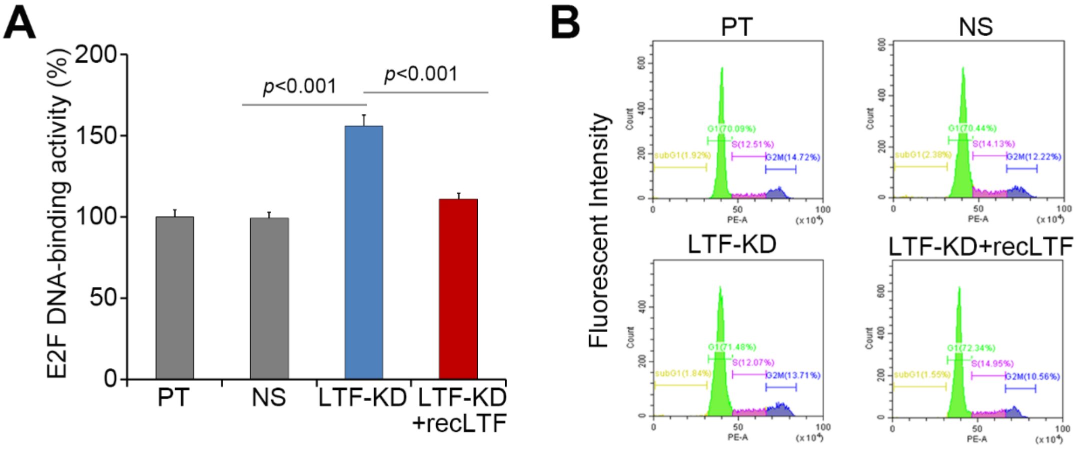

Figure S3. LTF effects on E2F DNA-binding activity and cell cycle progression in A498 cells. E2F DNA-binding activity determined by luciferase-based reporter assay (A) and cell counts in Sub-G1, Go/G1, S and G2M phases (B) measured by propidium iodide-based flow cytometric analysis in parental (PT) and non-silencing (NS) control A498 cells and LTF-silencing (LTF knockdown, LTF-KD) A498 cells pretreated without or with recLTF protein at 300 ng/ml for 24 hours. One-way ANOVA with Tukey's test was used to estimate statistical significance in A.

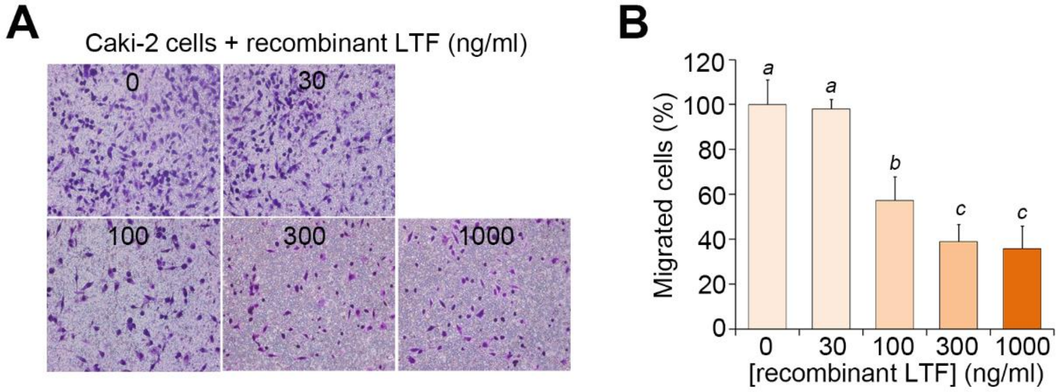

Figure S4. LTF inhibits cellular migration ability in Caki2 cells. (A and B) Giemsa staining (A) and histogram representing data from three independent experiments (B) analyzing migrated cells in the 3-hour Transwell assay for Caki2 cells pretreated with recombinant LTF protein at the indicated concentrations for 24 hours. Statistical significance was determined by the Friedman test.

Uncut Blots:

Fgure S2 blots

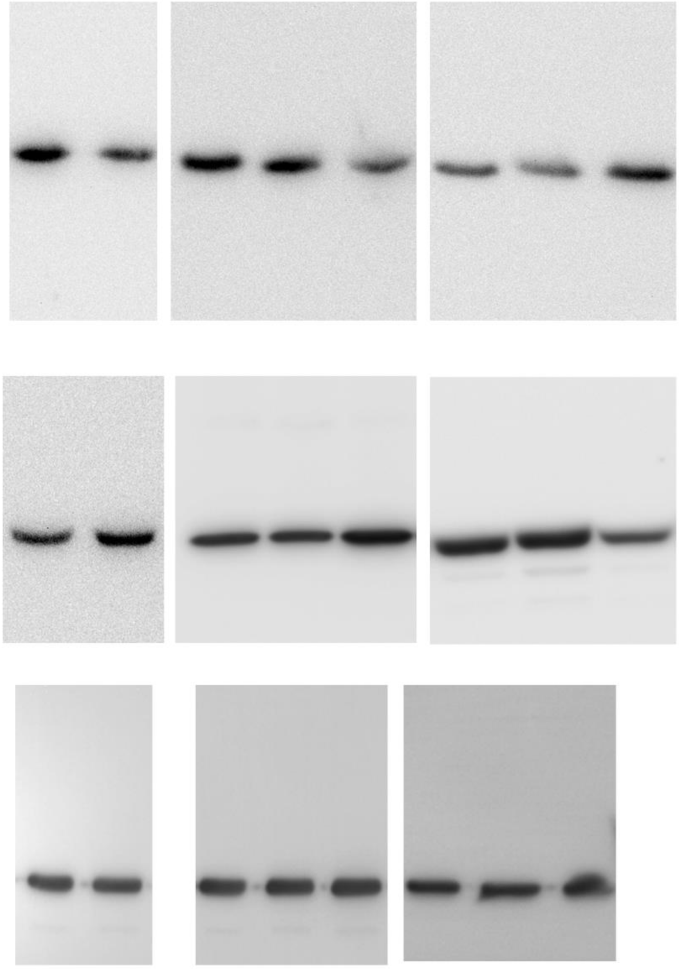

Fgure 6A blots

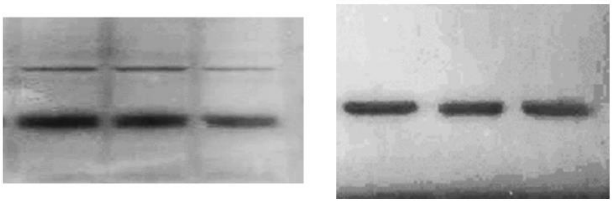

Table S1. Clincal data of TCGA ccRCC patients analyzed in this study.

sample	age	gender	stage	grade	OS	OS_time	LTF	LTF_spss
TCGA-3Z-A93Z-01	69	MALE	Stage I	G2	0	12.66	-0.6	2
TCGA-6D-AA2E-01	68	FEMALE	Stage I	G2	0	11.90	-1.66	2
TCGA-A3-3306-01	67	MALE	Stage I	G3	0	36.82	-2.96	2
TCGA-A3-3307-01	66	MALE	Stage III	G3	0	47.21	-2.3	2
TCGA-A3-3308-01	77	FEMALE	Stage III	G2	0	0.53	1.17	2
TCGA-A3-3311-01	57	MALE	Stage I	G2	1	39.16	-1.14	2
TCGA-A3-3313-01	59	MALE	Stage I	G3	1	24.16	-1.6	2
TCGA-A3-3316-01	57	MALE	Stage II	G3	0	49.08	-1.25	2
TCGA-A3-3317-01	67	MALE	Stage II	G2	0	49.02	1.323	2
TCGA-A3-3319-01	70	MALE	Stage I	G2	0	37.15	1.032	2
TCGA-A3-3320-01	52	FEMALE	Stage I	G1	0	49.58	-1.93	2
TCGA-A3-3322-01	51	MALE	Stage I	G2	0	48.59	-1.23	2
TCGA-A3-3323-01	53	MALE	Stage I	G1	0	36.36	4.804	2
TCGA-A3-3324-01	51	MALE	Stage I	G3	0	38.99	-3.03	2
TCGA-A3-3325-01	52	MALE	Stage I	G2	1	38.47	-0.37	2
TCGA-A3-3326-01	47	MALE	Stage I	G1	0	37.38	-0.39	2
TCGA-A3-3328-01	79	MALE	Stage I	G2	0	45.53	1.597	2
TCGA-A3-3329-01	75	MALE	Stage I	G2	0	53.39	-3.49	2
TCGA-A3-3331-01	86	FEMALE	Stage I	G2	0	48.82	-1.02	2
TCGA-A3-3335-01	41	MALE	Stage II	G4	0	62.01	5.213	2
TCGA-A3-3343-01	79	MALE	Stage II	G3	0	31.07	-3.01	2
TCGA-A3-3346-01	68	MALE	Stage I	G3	1	4.50	-1.89	2
TCGA-A3-3347-01	76	FEMALE	Stage III	G2	1	52.93	1.115	2
TCGA-A3-3349-01	34	FEMALE	Stage I	G2	0	45.53	2.936	2
TCGA-A3-3351-01	42	MALE	Stage II	G2	0	29.92	5.725	2
TCGA-A3-3352-01	74	MALE	Stage III	G3	1	18.44	-2.91	2
TCGA-A3-3357-01	62	MALE	Stage II	G3	0	88.37	-1.6	2
TCGA-A3-3358-01	57	FEMALE	Stage I	G2	0	42.97	3.241	2
TCGA-A3-3359-01	82	FEMALE	Stage I	G2	0	82.32	-1.05	2
TCGA-A3-3362-01	60	FEMALE	Stage I	G2	0	51.25	2.539	2
TCGA-A3-3363-01	50	MALE	Stage II	G2	0	10.49	0.116	2
TCGA-A3-3365-01	46	MALE	Stage I	G2	0	28.70	0.336	2
TCGA-A3-3367-01	72	MALE	Stage I	G3	0	74.63	-2.08	2
TCGA-A3-3370-01	48	FEMALE	Stage I	G2	0	74.76	-1.41	2
TCGA-A3-3372-01	64	MALE	Stage III	G2	0	24.16	-2.91	2
TCGA-A3-3373-01	54	FEMALE	Stage I	G3	0	53.29	-1.67	2
TCGA-A3-3374-01	51	FEMALE	Stage I	G2	0	43.20	2.811	2
TCGA-A3-3376-01	51	MALE	Stage I	G2	1	55.76	-0.3	2
TCGA-A3-3378-01	60	MALE	Stage I	G3	0	20.71	5.285	2
TCGA-A3-3380-01	54	MALE	Stage I	G2	0	18.64	-1.12	2
TCGA-A3-3382-01	69	MALE	Stage I	G3	0	18.87	-4.01	2
TCGA-A3-3383-01	52	MALE	Stage I	G2	0	28.31	-1.47	2
TCGA-A3-3385-01	46	FEMALE	Stage I	G2	0	65.52	-3.37	2
TCGA-A3-3387-01	49	MALE	Stage I	G2	0	20.28	-0.04	2

TCGA-A3-A6NI-01	47	MALE	Stage I	G3	0	33.47	-1.95	2
TCGA-A3-A6NJ-01	57	FEMALE	Stage I	G1	0	15.39	-0.78	2
TCGA-A3-A6NL-01	49	FEMALE	Stage I	G2	0	22.65	1.281	2
TCGA-A3-A6NN-01	78	MALE	Stage I	G2	0	0.10	-1.41	2
TCGA-A3-A8CQ-01	59	FEMALE	Stage I	G2	0	0.10	-0.25	2
TCGA-A3-A8OV-01	75	MALE	Stage I	G2	0	11.18	-0.74	2
TCGA-A3-A8OW-01	37	MALE	Stage III	G2	0	10.62	-0.97	2
TCGA-AK-3425-01	68	MALE	Stage I	G2	0	109.91	0.441	2
TCGA-AK-3426-01	37	MALE	Stage III	G3	1	29.10	-1.31	2
TCGA-AK-3428-01	62	MALE	Stage III	G2	0	122.56	0.69	2
TCGA-AK-3429-01	54	FEMALE	Stage II	G2	0	109.41	-1.7	2
TCGA-AK-3431-01	62	FEMALE	Stage II	G3	1	73.68	-0.72	2
TCGA-AK-3434-01	72	MALE	Stage I	G2	0	68.61	-0.93	2
TCGA-AK-3436-01	40	MALE	Stage IV	G2	0	109.51	-3.58	2
TCGA-AK-3440-01	58	MALE	Stage I	G3	0	94.19	0.946	2
TCGA-AK-3444-01	80	FEMALE	Stage I	G2	0	48.36	-1.03	2
TCGA-AK-3445-01	69	MALE	Stage III	G3	0	78.64	-2.28	2
TCGA-AK-3447-01	83	MALE	Stage II	G2	0	40.01	-1.76	2
TCGA-AK-3450-01	85	FEMALE	Stage I	G2	0	58.49	-1.14	2
TCGA-AK-3451-01	48	MALE	Stage II	G3	0	94.29	-0.23	2
TCGA-AK-3453-01	58	FEMALE	Stage II	G2	0	83.21	-1.77	2
TCGA-AK-3454-01	84	MALE	Stage I	G3	0	28.73	-3.47	2
TCGA-AK-3455-01	71	FEMALE	Stage III	G3	1	22.45	-3.76	2
TCGA-AK-3456-01	48	MALE	Stage II	G3	0	37.58	2.161	2
TCGA-AK-3458-01	48	MALE	Stage I	G3	0	38.40	-1.28	2
TCGA-AK-3460-01	58	MALE	Stage I	G2	0	82.45	-0.19	2
TCGA-AK-3461-01	72	MALE	Stage I	G2	0	72.89	0.933	2
TCGA-AS-3778-01	35	MALE	Stage I	G1	0	1.41	3.958	2
TCGA-B0-4688-01	46	MALE	Stage IV	G4	1	3.32	-4.41	1
TCGA-B0-4690-01	65	MALE	Stage IV	G3	1	1.41	3.278	2
TCGA-B0-4691-01	55	MALE	Stage IV	G3	1	4.57	0.642	2
TCGA-B0-4693-01	72	FEMALE	Stage III	G4	1	2.53	-1.38	2
TCGA-B0-4694-01	72	MALE	Stage III	G4	1	3.48	4.417	2
TCGA-B0-4696-01	58	MALE	Stage III	G3	1	28.47	-3.79	2
TCGA-B0-4697-01	46	FEMALE	Stage IV	G4	1	19.00	-0.16	2
TCGA-B0-4698-01	75	MALE	Stage IV	G4	1	1.38	-3.49	2
TCGA-B0-4699-01	74	MALE	Stage IV	G4	1	3.62	1.58	2
TCGA-B0-4700-01	60	MALE	Stage IV	G4	1	65.10	1.494	2
TCGA-B0-4701-01	66	FEMALE	Stage IV	G3	1	7.82	-2.9	2
TCGA-B0-4703-01	51	MALE	Stage IV	G4	1	5.98	-0.44	2
TCGA-B0-4706-01	61	MALE	Stage III	G4	1	2.14	0.309	2
TCGA-B0-4707-01	63	MALE	Stage III	G4	1	19.73	0.061	2
TCGA-B0-4710-01	75	FEMALE	Stage III	G3	0	57.70	2.287	2
TCGA-B0-4712-01	76	MALE	Stage IV	G3	1	43.96	-0.55	2
TCGA-B0-4713-01	76	FEMALE	Stage III	G2	1	6.64	0.047	2
TCGA-B0-4714-01	81	MALE	Stage IV	G3	1	3.25	-1.8	2

TCGA-B0-4718-01	57	MALE	Stage III	G2	0	58.45	-1.84	2
TCGA-B0-4810-01	47	MALE	Stage III	G3	1	15.72	3.169	2
TCGA-B0-4811-01	48	MALE	Stage III	G3	1	46.59	1.261	2
TCGA-B0-4813-01	68	MALE	Stage III	G3	1	0.59	-1.34	2
TCGA-B0-4814-01	58	MALE	Stage IV	G3	1	5.52	-2.69	2
TCGA-B0-4815-01	65	MALE	Stage III	G4	1	52.21	-1.56	2
TCGA-B0-4816-01	49	MALE	Stage II	G3	1	45.07	0.258	2
TCGA-B0-4817-01	81	MALE	Stage III	G3	1	33.50	3.686	2
TCGA-B0-4818-01	68	FEMALE	Stage II	G3	1	16.77	-1.22	2
TCGA-B0-4819-01	60	FEMALE	Stage IV	G4	1	6.02	3.083	2
TCGA-B0-4821-01	68	FEMALE	Stage III	G3	1	40.44	-5.43	1
TCGA-B0-4822-01	78	MALE	Stage II	G4	1	36.53	-0.69	2
TCGA-B0-4823-01	88	MALE	Stage I	G2	1	14.93	0.365	2
TCGA-B0-4824-01	49	FEMALE	Stage I	G3	1	54.48	-2.13	2
TCGA-B0-4827-01	77	FEMALE	Stage III	G4	1	29.10	6.053	2
TCGA-B0-4828-01	79	MALE	Stage IV	G3	1	10.09	-3.06	2
TCGA-B0-4833-01	82	FEMALE	Stage I	G2	1	78.44	-1.27	2
TCGA-B0-4834-01	49	MALE	Stage I	G3	1	68.71	2.988	2
TCGA-B0-4836-01	61	MALE	Stage IV	G3	1	40.70	-0.3	2
TCGA-B0-4837-01	63	MALE	Stage I	G3	1	45.30	-3.27	2
TCGA-B0-4838-01	69	FEMALE	Stage I	G3	1	27.42	-2.2	2
TCGA-B0-4839-01	80	FEMALE	Stage I	G2	1	53.88	6.267	2
TCGA-B0-4841-01	63	MALE	Stage IV	G3	1	6.71	0.47	2
TCGA-B0-4842-01	73	FEMALE	Stage III	G4	1	56.68	-0.27	2
TCGA-B0-4843-01	57	MALE	Stage III	G3	1	10.52	7.208	2
TCGA-B0-4844-01	60	MALE	Stage IV	G3	1	10.29	2.009	2
TCGA-B0-4845-01	70	MALE	Stage IV	G2	1	65.29	-1.14	2
TCGA-B0-4846-01	52	MALE	Stage IV	G2	1	39.45	-1.88	2
TCGA-B0-4847-01	60	MALE	Stage IV	G3	1	26.07	-2.12	2
TCGA-B0-4848-01	54	MALE	Stage III	G3	1	29.03	1.011	2
TCGA-B0-4849-01	51	MALE	Stage III	G3	1	2.27	-2.88	2
TCGA-B0-4852-01	78	FEMALE	Stage II	G2	1	36.85	1.183	2
TCGA-B0-4945-01	75	FEMALE	Stage I	G2	1	70.52	-0.52	2
TCGA-B0-5075-01	77	FEMALE	Stage III	G2	1	20.94	-2.37	2
TCGA-B0-5077-01	77	MALE	Stage I	G3	1	43.30	-0.73	2
TCGA-B0-5080-01	63	MALE	Stage IV	G3	1	11.24	0.569	2
TCGA-B0-5081-01	79	FEMALE	Stage III	G2	1	11.90	-0.36	2
TCGA-B0-5083-01	63	MALE	Stage I	G3	1	34.36	3.692	2
TCGA-B0-5084-01	33	MALE	Stage IV	G3	1	7.30	-2.82	2
TCGA-B0-5085-01	76	FEMALE	Stage III	G3	1	25.32	-1.01	2
TCGA-B0-5088-01	53	MALE	Stage I	G3	1	18.51	0.932	2
TCGA-B0-5092-01	53	FEMALE	Stage IV	G3	1	15.09	1.869	2
TCGA-B0-5094-01	62	MALE	Stage IV	G2	1	10.95	1.347	2
TCGA-B0-5095-01	81	MALE	Stage III	G3	1	8.05	-0.76	2
TCGA-B0-5097-01	59	FEMALE	Stage III	G2	0	21.86	-1.79	2
TCGA-B0-5098-01	53	FEMALE	Stage I	G3	1	52.08	-0.1	2

TCGA-B0-5099-01	88	FEMALE	Stage III	G3	1	15.95	-2.23	2
TCGA-B0-5100-01	72	MALE	Stage III	G3	1	62.89	4.603	2
TCGA-B0-5102-01	74	FEMALE	Stage I	G3	1	90.87	3.705	2
TCGA-B0-5104-01	90	FEMALE	Stage I	G2	1	90.48	-1.6	2
TCGA-B0-5106-01	64	MALE	Stage I	G2	1	52.54	3.72	2
TCGA-B0-5107-01	65	FEMALE	Stage IV	G4	1	30.48	-5.79	1
TCGA-B0-5108-01	54	MALE	Stage III	G2	0	58.59	3.47	2
TCGA-B0-5109-01	69	MALE	Stage III	G4	1	19.30	5.743	2
TCGA-B0-5110-01	71	FEMALE	Stage I	G2	0	66.05	-1.33	2
TCGA-B0-5113-01	69	FEMALE	Stage III	G2	0	38.63	-2.65	2
TCGA-B0-5115-01	43	MALE	Stage IV	G3	0	52.73	-0.6	2
TCGA-B0-5116-01	52	MALE	Stage III	G3	0	41.88	-1.78	2
TCGA-B0-5117-01	40	MALE	Stage I	G2	0	52.87	2.658	2
TCGA-B0-5119-01	61	FEMALE	Stage I	G2	0	51.02	-0.37	2
TCGA-B0-5120-01	72	FEMALE	Stage I	G2	0	38.43	-0.1	2
TCGA-B0-5121-01	56	MALE	Stage I	G2	0	48.82	-0.86	2
TCGA-B0-5399-01	46	MALE	Stage I	G2	0	46.39	-1.06	2
TCGA-B0-5400-01	59	FEMALE	Stage III	G4	0	56.98	1.069	2
TCGA-B0-5402-01	64	MALE	Stage IV	G4	0	42.41	-1.84	2
TCGA-B0-5690-01	53	FEMALE	Stage I	G1	0	111.52	-1.34	2
TCGA-B0-5691-01	66	FEMALE	Stage I	G3	0	112.80	1.992	2
TCGA-B0-5692-01	66	FEMALE	Stage III	G3	0	129.67	-2.88	2
TCGA-B0-5693-01	47	FEMALE	Stage I	G2	0	133.94	-0.81	2
TCGA-B0-5694-01	71	MALE	Stage III	G3	1	15.78	0.069	2
TCGA-B0-5695-01	61	FEMALE	Stage I	G2	0	70.68	2.333	2
TCGA-B0-5696-01	69	MALE	Stage III	G4	0	85.78	2.365	2
TCGA-B0-5697-01	50	MALE	Stage I	G2	0	86.47	0.087	2
TCGA-B0-5698-01	77	MALE	Stage I	G3	0	119.38	-4.01	2
TCGA-B0-5699-01	53	MALE	Stage I	G2	0	126.28	-1.83	2
TCGA-B0-5700-01	77	MALE	Stage I	G2	0	58.85	-0.43	2
TCGA-B0-5701-01	65	MALE	Stage III	G4	0	80.91	-2.08	2
TCGA-B0-5702-01	71	MALE	Stage I	G2	0	71.41	4.426	2
TCGA-B0-5703-01	73	MALE	Stage I	G3	0	73.84	-3.19	2
TCGA-B0-5705-01	65	FEMALE	Stage I	G2	0	149.16	-0.68	2
TCGA-B0-5706-01	45	MALE	Stage II	G2	0	105.37	5.998	2
TCGA-B0-5707-01	39	FEMALE	Stage I	G3	0	123.09	1.015	2
TCGA-B0-5709-01	62	FEMALE	Stage III	G3	0	130.65	1.252	2
TCGA-B0-5710-01	57	MALE	Stage I	G2	0	79.89	-2.73	2
TCGA-B0-5711-01	50	MALE	Stage III	G3	0	131.15	-2.34	2
TCGA-B0-5712-01	68	FEMALE	Stage IV	G3	0	89.49	1.989	2
TCGA-B0-5713-01	75	FEMALE	Stage III	G3	0	91.46	-1.44	2
TCGA-B0-5812-01	53	MALE	Stage I	G3	0	126.05	1.896	2
TCGA-B2-3923-01	59	MALE	Stage II	G2	0	32.61	3.124	2
TCGA-B2-3924-01	73	MALE	Stage I	G2	0	35.90	-0.31	2
TCGA-B2-4098-01	72	FEMALE	Stage I	G2	1	1.68	0.769	2
TCGA-B2-4099-01	83	MALE	Stage I	G3	0	31.96	-1.27	2

TCGA-B2-4101-01	52	MALE	Stage II	G3	0	21.30	-1.21	2
TCGA-B2-4102-01	61	MALE	Stage I	G2	0	31.30	0.53	2
TCGA-B2-5633-01	56	MALE	Stage I	G2	0	31.66	3.017	2
TCGA-B2-5635-01	74	MALE	Stage I	G2	0	24.82	5E-04	2
TCGA-B2-5636-01	79	MALE	Stage I	G2	0	30.21	3.605	2
TCGA-B2-5639-01	46	MALE	Stage IV	G3	1	32.98	-0.52	2
TCGA-B2-5641-01	79	MALE	Stage I	G3	0	21.57	8.472	2
TCGA-B4-5377-01	68	FEMALE	Stage IV	G3	0	12.00	1.217	2
TCGA-B4-5378-01	62	MALE	Stage I	G2	0	5.75	0.133	2
TCGA-B4-5832-01	65	MALE	Stage III	G2	0	5.10	-2.58	2
TCGA-B4-5834-01	59	MALE	Stage I	G1	0	0.85	-1.28	2
TCGA-B4-5835-01	64	FEMALE	Stage I	G2	0	0.53	1.368	2
TCGA-B4-5836-01	61	FEMALE	Stage I	G2	0	4.64	1.394	2
TCGA-B4-5838-01	52	MALE	Stage IV	G2	0	5.46	2.949	2
TCGA-B4-5843-01	45	MALE	Stage I	G2	0	0.36	-0.68	2
TCGA-B4-5844-01	61	FEMALE	Stage II	G1	0	0.23	-1.8	2
TCGA-B8-4143-01	66	FEMALE	Stage IV	G3	1	23.31	-3.89	2
TCGA-B8-4146-01	41	FEMALE	Stage I	G2	0	16.80	-0.04	2
TCGA-B8-4148-01	63	FEMALE	Stage I	G3	0	49.97	-2.12	2
TCGA-B8-4151-01	51	FEMALE	Stage III	G2	0	42.71	-2.9	2
TCGA-B8-4153-01	74	MALE	Stage III	G3	0	25.05	-2.39	2
TCGA-B8-4154-01	73	FEMALE	Stage I	G2	0	45.37	-1.15	2
TCGA-B8-4619-01	58	MALE	Stage I	G2	0	17.19	2.339	2
TCGA-B8-4620-01	70	FEMALE	Stage III	G2	0	25.55	-1.37	2
TCGA-B8-4621-01	63	MALE	Stage I	G3	0	25.91	1.484	2
TCGA-B8-4622-01	57	MALE	Stage IV	G3	0	50.14	-2.74	2
TCGA-B8-5158-01	56	MALE	Stage III	G4	0	40.04	1.513	2
TCGA-B8-5159-01	61	FEMALE	Stage I	G3	0	23.74	-2.03	2
TCGA-B8-5162-01	62	MALE	Stage II	G2	0	1.18	-0.93	2
TCGA-B8-5163-01	63	FEMALE	Stage III	G3	0	27.02	-3.42	2
TCGA-B8-5164-01	65	MALE	Stage III	G3	0	0.85	1.247	2
TCGA-B8-5165-01	43	MALE	Stage I	G2	0	24.23	1.631	2
TCGA-B8-5545-01	42	MALE	Stage I	G2	0	50.14	1.909	2
TCGA-B8-5546-01	38	FEMALE	Stage I	G2	0	16.60	-0.73	2
TCGA-B8-5549-01	53	MALE	Stage I	G3	0	6.38	2.488	2
TCGA-B8-5550-01	71	MALE	Stage III	G3	0	48.53	1.987	2
TCGA-B8-5551-01	65	FEMALE	Stage I	G3	0	0.53	0.416	2
TCGA-B8-5552-01	41	FEMALE	Stage I	G2	0	34.39	4.821	2
TCGA-B8-5553-01	67	FEMALE	Stage I	G2	0	14.30	2.349	2
TCGA-B8-A54D-01	69	MALE	Stage III	G2	0	27.29	-2.79	2
TCGA-B8-A54E-01	62	FEMALE	Stage I	G3	0	29.88	-4.37	2
TCGA-B8-A54F-01	49	FEMALE	Stage I	G2	0	17.06	0.581	2
TCGA-B8-A54G-01	50	MALE	Stage I	G3	0	1.74	0.67	2
TCGA-B8-A54H-01	69	FEMALE	Stage II	G3	0	8.42	-1.71	2
TCGA-B8-A54I-01	48	MALE	Stage I	G3	0	4.93	5.109	2
TCGA-B8-A54J-01	60	MALE	Stage II	G2	0	17.36	-0.29	2

TCGA-B8-A54K-01	61	MALE	Stage I	G1	0	15.42	1.83	2
TCGA-BP-4158-01	69	MALE	Stage I	G2	0	111.02	-2.02	2
TCGA-BP-4159-01	70	MALE	Stage I	G2	1	85.51	0.688	2
TCGA-BP-4160-01	67	MALE	Stage III	G2	0	94.72	-1.23	2
TCGA-BP-4161-01	74	MALE	Stage I	G3	0	90.28	-1.36	2
TCGA-BP-4162-01	65	FEMALE	Stage I	G2	0	101.06	-2.65	2
TCGA-BP-4163-01	60	FEMALE	Stage III	G3	0	93.34	3.592	2
TCGA-BP-4164-01	51	FEMALE	Stage III	G2	1	32.61	-4.88	1
TCGA-BP-4165-01	64	FEMALE	Stage I	G1	0	99.85	-1.93	2
TCGA-BP-4166-01	69	MALE	Stage III	G3	0	0.43	-1.82	2
TCGA-BP-4167-01	59	MALE	Stage III	G2	0	89.36	-0.78	2
TCGA-BP-4169-01	76	FEMALE	Stage II	G2	1	23.05	1.087	2
TCGA-BP-4170-01	72	FEMALE	Stage I	G2	1	77.03	-1.48	2
TCGA-BP-4173-01	47	MALE	Stage II	G3	0	62.24	8.502	2
TCGA-BP-4174-01	49	MALE	Stage II	G3	0	61.78	2.445	2
TCGA-BP-4176-01	64	MALE	Stage I	G2	0	64.27	-1.46	2
TCGA-BP-4177-01	65	MALE	Stage I	G2	0	54.90	4.163	2
TCGA-BP-4325-01	64	FEMALE	Stage I	G2	0	97.45	-2.28	2
TCGA-BP-4326-01	53	FEMALE	Stage I	G2	1	53.42	3.656	2
TCGA-BP-4327-01	75	FEMALE	Stage II	G2	1	3.58	2.709	2
TCGA-BP-4329-01	75	MALE	Stage III	G2	1	27.78	-0.61	2
TCGA-BP-4330-01	60	FEMALE	Stage III	G2	0	62.07	-0.59	2
TCGA-BP-4331-01	52	MALE	Stage I	G2	1	80.68	1.032	2
TCGA-BP-4332-01	36	MALE	Stage III	G2	0	37.25	3.798	2
TCGA-BP-4334-01	56	MALE	Stage III	G3	1	21.21	-0.27	2
TCGA-BP-4335-01	65	FEMALE	Stage IV	G3	1	15.62	-0.97	2
TCGA-BP-4337-01	76	FEMALE	Stage III	G4	1	0.07	1.397	2
TCGA-BP-4338-01	43	MALE	Stage I	G3	0	93.99	-4.11	2
TCGA-BP-4340-01	70	FEMALE	Stage I	G2	1	18.48	-2.25	2
TCGA-BP-4341-01	67	MALE	Stage III	G2	1	52.24	-1.53	2
TCGA-BP-4342-01	79	MALE	Stage II	G3	1	74.17	-1.29	2
TCGA-BP-4343-01	64	MALE	Stage III	G3	1	62.86	0.782	2
TCGA-BP-4344-01	75	FEMALE	Stage I	G2	0	54.77	0.015	2
TCGA-BP-4345-01	62	MALE	Stage III	G3	0	49.84	-0.2	2
TCGA-BP-4346-01	57	MALE	Stage III	G3	1	49.08	-1.81	2
TCGA-BP-4347-01	74	MALE	Stage III	G2	0	44.94	-1.46	2
TCGA-BP-4349-01	68	FEMALE	Stage I	G2	0	12.23	-1.93	2
TCGA-BP-4351-01	51	FEMALE	Stage III	G2	0	31.89	-3.05	2
TCGA-BP-4352-01	74	FEMALE	Stage IV	G4	1	11.31	-4.24	2
TCGA-BP-4353-01	61	MALE	Stage I	G2	1	12.33	-0.89	2
TCGA-BP-4354-01	40	MALE	Stage IV	G4	1	33.99	-3.45	2
TCGA-BP-4355-01	59	FEMALE	Stage III	G4	1	31.33	-1.56	2
TCGA-BP-4756-01	62	FEMALE	Stage I	G2	0	12.30	-1.31	2
TCGA-BP-4758-01	40	MALE	Stage I	G2	0	72.59	0.419	2
TCGA-BP-4759-01	50	MALE	Stage I	G2	0	77.98	-1.63	2
TCGA-BP-4760-01	69	MALE	Stage I	G2	0	77.62	2.679	2

TCGA-BP-4761-01	57	MALE	Stage III	G4	0	5.98	-1.49	2
TCGA-BP-4762-01	42	MALE	Stage I	G3	1	44.15	3.822	2
TCGA-BP-4763-01	79	FEMALE	Stage I	G2	1	41.75	0.858	2
TCGA-BP-4765-01	43	MALE	Stage I	G2	0	71.80	0.084	2
TCGA-BP-4766-01	43	FEMALE	Stage I	G3	0	48.07	-0.77	2
TCGA-BP-4768-01	72	FEMALE	Stage I	G2	0	13.15	-4.83	1
TCGA-BP-4769-01	63	MALE	Stage I	G2	0	61.68	8.882	2
TCGA-BP-4770-01	73	FEMALE	Stage IV	G4	1	10.82	-5.67	1
TCGA-BP-4771-01	62	MALE	Stage IV	G4	1	5.33	3.469	2
TCGA-BP-4774-01	57	FEMALE	Stage I	G2	0	61.97	-0.96	2
TCGA-BP-4775-01	55	FEMALE	Stage I	G2	0	60.59	-1.9	2
TCGA-BP-4776-01	52	MALE	Stage I	G2	0	13.51	-0.42	2
TCGA-BP-4777-01	46	MALE	Stage I	G3	0	56.91	0.061	2
TCGA-BP-4781-01	78	MALE	Stage I	G3	0	68.38	-3.01	2
TCGA-BP-4782-01	55	FEMALE	Stage I	G2	0	11.64	-0.29	2
TCGA-BP-4784-01	67	FEMALE	Stage I	G2	0	60.95	5.815	2
TCGA-BP-4787-01	59	FEMALE	Stage IV	G4	1	15.78	-2.04	2
TCGA-BP-4789-01	48	MALE	Stage I	G2	0	48.95	-0.51	2
TCGA-BP-4790-01	76	MALE	Stage I	G2	1	36.53	4.004	2
TCGA-BP-4795-01	74	FEMALE	Stage I	G2	0	20.38	4.939	2
TCGA-BP-4797-01	34	MALE	Stage III	G3	0	36.39	5.674	2
TCGA-BP-4799-01	70	MALE	Stage III	G3	1	37.25	-2.24	2
TCGA-BP-4801-01	57	MALE	Stage I	G2	0	36.95	1.148	2
TCGA-BP-4803-01	79	MALE	Stage III	G3	0	6.71	-1.85	2
TCGA-BP-4804-01	59	MALE	Stage I	G2	0	47.97	2.977	2
TCGA-BP-4807-01	42	MALE	Stage I	G3	0	6.94	1.788	2
TCGA-BP-4959-01	49	MALE	Stage I	G3	0	87.45	6.85	2
TCGA-BP-4960-01	46	MALE	Stage II	G3	0	71.41	5.283	2
TCGA-BP-4961-01	47	MALE	Stage I	G2	0	63.62	-0.31	2
TCGA-BP-4962-01	58	MALE	Stage II	G2	0	58.68	-0.73	2
TCGA-BP-4963-01	63	MALE	Stage I	G3	0	60.30	-3.02	2
TCGA-BP-4964-01	54	FEMALE	Stage I	G2	0	61.22	-1.51	2
TCGA-BP-4965-01	46	MALE	Stage I	G2	0	61.51	-0.3	2
TCGA-BP-4967-01	76	MALE	Stage III	G2	0	6.74	3.122	2
TCGA-BP-4968-01	40	MALE	Stage I	G3	0	57.40	-0.42	2
TCGA-BP-4969-01	63	FEMALE	Stage I	G2	0	58.98	-0.73	2
TCGA-BP-4970-01	44	MALE	Stage III	G3	0	14.24	5.262	2
TCGA-BP-4971-01	40	MALE	Stage III	G3	0	48.89	0.042	2
TCGA-BP-4972-01	43	FEMALE	Stage III	G3	0	49.38	-1.01	2
TCGA-BP-4973-01	47	MALE	Stage III	G3	0	45.50	-0.24	2
TCGA-BP-4974-01	58	MALE	Stage IV	G4	1	6.94	1.068	2
TCGA-BP-4975-01	40	MALE	Stage I	G3	0	47.11	0.278	2
TCGA-BP-4976-01	77	MALE	Stage I	G3	0	53.65	2.981	2
TCGA-BP-4977-01	57	MALE	Stage I	G3	0	14.93	0.241	2
TCGA-BP-4981-01	75	FEMALE	Stage III	G3	1	36.07	-1.28	2
TCGA-BP-4982-01	42	MALE	Stage I	G3	0	33.34	-0.41	2

TCGA-BP-4983-01	67	FEMALE	Stage III	G4	0	46.45	1.12	2
TCGA-BP-4985-01	72	MALE	Stage III	G4	1	31.30	-4.1	2
TCGA-BP-4986-01	75	MALE	Stage I	G3	0	25.81	-2.14	2
TCGA-BP-4987-01	41	FEMALE	Stage I	G2	0	36.95	-0.75	2
TCGA-BP-4988-01	72	MALE	Stage I	G2	1	27.22	1.058	2
TCGA-BP-4989-01	58	MALE	Stage III	G3	0	3.88	-2.02	2
TCGA-BP-4991-01	54	MALE	Stage I	G2	0	46.45	3.45	2
TCGA-BP-4992-01	66	MALE	Stage I	G4	0	16.47	5.075	2
TCGA-BP-4993-01	58	MALE	Stage I	G3	0	5.82	1.77	2
TCGA-BP-4994-01	54	MALE	Stage I	G3	0	43.00	2.479	2
TCGA-BP-4995-01	68	MALE	Stage I	G3	0	45.07	1.91	2
TCGA-BP-4998-01	49	MALE	Stage I	G3	0	30.64	5.372	2
TCGA-BP-4999-01	56	MALE	Stage I	G2	0	41.62	-1.3	2
TCGA-BP-5000-01	40	MALE	Stage I	G3	0	18.51	7.552	2
TCGA-BP-5001-01	43	FEMALE	Stage I	G2	0	38.70	-0.87	2
TCGA-BP-5004-01	53	MALE	Stage I	G3	0	37.02	-1.32	2
TCGA-BP-5006-01	61	MALE	Stage I	G2	0	27.62	-0.91	2
TCGA-BP-5007-01	45	MALE	Stage II	G2	0	37.48	-0.7	2
TCGA-BP-5008-01	46	MALE	Stage I	G2	0	35.21	3.592	2
TCGA-BP-5009-01	52	MALE	Stage I	G3	1	35.90	-2.18	2
TCGA-BP-5010-01	63	MALE	Stage III	G4	1	28.87	-1.28	2
TCGA-BP-5168-01	75	MALE	Stage I	G2	1	48.10	-1.58	2
TCGA-BP-5169-01	70	MALE	Stage I	G4	0	6.35	0.025	2
TCGA-BP-5170-01	55	MALE	Stage I	G2	0	79.30	0.076	2
TCGA-BP-5173-01	75	MALE	Stage I	G2	1	2.04	-2.11	2
TCGA-BP-5174-01	45	FEMALE	Stage I	G2	0	74.20	-0.89	2
TCGA-BP-5175-01	60	MALE	Stage I	G3	0	30.64	8.534	2
TCGA-BP-5176-01	78	FEMALE	Stage I	G2	1	52.27	-0.46	2
TCGA-BP-5177-01	46	FEMALE	Stage I	G3	0	9.63	-1.11	2
TCGA-BP-5178-01	71	MALE	Stage IV	G4	1	62.86	-2.15	2
TCGA-BP-5180-01	53	MALE	Stage I	G2	0	74.40	2.853	2
TCGA-BP-5181-01	58	FEMALE	Stage I	G2	0	49.15	-2.52	2
TCGA-BP-5182-01	56	MALE	Stage I	G3	0	38.30	0.126	2
TCGA-BP-5183-01	57	MALE	Stage III	G3	0	42.44	-2.11	2
TCGA-BP-5184-01	54	MALE	Stage I	G3	0	37.25	-0.09	2
TCGA-BP-5185-01	56	MALE	Stage I	G3	0	37.22	-1.44	2
TCGA-BP-5186-01	50	FEMALE	Stage I	G2	0	22.78	-0.31	2
TCGA-BP-5187-01	54	MALE	Stage I	G2	0	13.35	-0.07	2
TCGA-BP-5189-01	60	MALE	Stage I	G4	1	27.02	0.657	2
TCGA-BP-5190-01	61	MALE	Stage I	G3	0	33.24	-3.09	2
TCGA-BP-5191-01	79	MALE	Stage III	G2	0	31.79	6.171	2
TCGA-BP-5192-01	59	MALE	Stage I	G2	0	23.47	0.004	2
TCGA-BP-5194-01	39	MALE	Stage I	G2	0	13.41	-1.49	2
TCGA-BP-5195-01	75	MALE	Stage I	G2	0	24.62	-1.11	2
TCGA-BP-5196-01	53	MALE	Stage I	G2	0	33.47	2.59	2
TCGA-BP-5198-01	72	MALE	Stage III	G3	0	19.82	-1.94	2

TCGA-BP-5199-01	58	MALE	Stage II	G4	0	44.55	-1.71	2
TCGA-BP-5200-01	44	MALE	Stage II	G4	0	34.95	-2.17	2
TCGA-BP-5201-01	63	MALE	Stage IV	G4	0	31.27	-0.83	2
TCGA-BP-5202-01	75	MALE	Stage III	G2	0	0.95	-1.17	2
TCGA-CJ-4634-01	60	FEMALE	Stage I	G2	0	115.00	-0.78	2
TCGA-CJ-4635-01	48	MALE	Stage I	G3	0	46.55	-3.34	2
TCGA-CJ-4636-01	51	MALE	Stage III	G3	0	63.25	1.21	2
TCGA-CJ-4637-01	52	FEMALE	Stage IV	G4	1	73.22	4.196	2
TCGA-CJ-4638-01	46	FEMALE	Stage IV	G4	1	14.17	-4.75	1
TCGA-CJ-4639-01	49	FEMALE	Stage II	G2	0	106.16	-2.59	2
TCGA-CJ-4640-01	49	MALE	Stage III	G4	0	114.41	6.841	2
TCGA-CJ-4641-01	55	FEMALE	Stage IV	G4	1	54.61	1.256	2
TCGA-CJ-4642-01	47	MALE	Stage II	G2	0	105.37	3.032	2
TCGA-CJ-4643-01	67	FEMALE	Stage II	G3	0	58.95	-1.74	2
TCGA-CJ-4644-01	48	FEMALE	Stage IV	G3	1	11.05	-1.78	2
TCGA-CJ-4868-01	42	MALE	Stage IV	G3	1	21.24	0.926	2
TCGA-CJ-4869-01	49	MALE	Stage III	G2	0	83.97	8.487	2
TCGA-CJ-4870-01	58	FEMALE	Stage III	G2	0	49.25	-1.27	2
TCGA-CJ-4871-01	63	MALE	Stage IV	G4	0	79.66	2.23	2
TCGA-CJ-4872-01	51	MALE	Stage I	G4	0	10.72	-0.17	2
TCGA-CJ-4873-01	85	FEMALE	Stage III	G3	0	74.27	-1.04	2
TCGA-CJ-4874-01	73	FEMALE	Stage I	G3	0	75.06	-2.07	2
TCGA-CJ-4875-01	67	MALE	Stage IV	G3	1	116.84	-1.94	2
TCGA-CJ-4876-01	57	MALE	Stage II	G3	0	64.27	-3.93	2
TCGA-CJ-4878-01	71	FEMALE	Stage III	G2	0	71.87	-2.19	2
TCGA-CJ-4881-01	41	MALE	Stage III	G3	0	66.21	1.326	2
TCGA-CJ-4882-01	57	MALE	Stage III	G3	0	61.91	1.971	2
TCGA-CJ-4884-01	72	FEMALE	Stage III	G3	0	57.83	-2.36	2
TCGA-CJ-4885-01	64	MALE	Stage IV	G3	0	113.46	-3.16	2
TCGA-CJ-4886-01	42	FEMALE	Stage I	G3	0	64.18	0.835	2
TCGA-CJ-4887-01	48	MALE	Stage IV	G3	1	30.64	0.056	2
TCGA-CJ-4888-01	59	MALE	Stage IV	G4	1	51.52	-0.04	2
TCGA-CJ-4889-01	63	FEMALE	Stage I	G4	0	63.98	2.276	2
TCGA-CJ-4890-01	72	MALE	Stage IV	G4	0	115.69	3.075	2
TCGA-CJ-4891-01	57	FEMALE	Stage III	G4	1	26.93	0.992	2
TCGA-CJ-4892-01	65	FEMALE	Stage I	G2	0	50.01	-0.72	2
TCGA-CJ-4893-01	76	FEMALE	Stage I	G3	0	24.66	-1.08	2
TCGA-CJ-4894-01	58	MALE	Stage III	G3	1	27.65	-2.06	2
TCGA-CJ-4895-01	62	MALE	Stage IV	G4	1	39.45	-1.35	2
TCGA-CJ-4897-01	79	FEMALE	Stage III	G3	0	109.84	-1.75	2
TCGA-CJ-4899-01	42	MALE	Stage I	G2	0	50.24	2.084	2
TCGA-CJ-4900-01	69	FEMALE	Stage IV	G4	1	56.35	-0.08	2
TCGA-CJ-4901-01	47	MALE	Stage III	G3	0	47.67	-2.05	2
TCGA-CJ-4902-01	61	MALE	Stage III	G3	0	49.97	-0.41	2
TCGA-CJ-4903-01	50	MALE	Stage I	G3	0	51.25	-1.08	2
TCGA-CJ-4904-01	60	FEMALE	Stage IV	G3	0	108.56	-1.96	2

TCGA-CJ-4905-01	62	FEMALE	Stage I	G2	0	49.18	-0.75	2
TCGA-CJ-4907-01	58	MALE	Stage III	G3	0	49.28	-0.4	2
TCGA-CJ-4908-01	38	MALE	Stage I	G2	0	50.33	-2.01	2
TCGA-CJ-4912-01	61	MALE	Stage II	G3	0	54.48	-1.54	2
TCGA-CJ-4916-01	69	FEMALE	Stage III	G3	0	45.14	-1.58	2
TCGA-CJ-4918-01	64	MALE	Stage IV	G4	1	3.06	-1.1	2
TCGA-CJ-4920-01	64	FEMALE	Stage I	G2	1	4.57	-3	2
TCGA-CJ-4923-01	63	FEMALE	Stage IV	G4	1	18.81	3.391	2
TCGA-CJ-5671-01	51	MALE	Stage I	G3	0	131.08	-1.39	2
TCGA-CJ-5672-01	84	MALE	Stage I	G3	1	72.00	4.965	2
TCGA-CJ-5675-01	70	MALE	Stage II	G3	0	129.40	-0.93	2
TCGA-CJ-5676-01	47	MALE	Stage III	G3	0	133.71	0.866	2
TCGA-CJ-5677-01	54	FEMALE	Stage IV	G4	1	25.71	2.029	2
TCGA-CJ-5678-01	62	MALE	Stage IV	G3	1	18.87	-3.19	2
TCGA-CJ-5679-01	73	MALE	Stage III	G4	1	22.32	-2.8	2
TCGA-CJ-5680-01	65	FEMALE	Stage IV	G4	1	25.25	0.15	2
TCGA-CJ-5681-01	44	FEMALE	Stage IV	G3	1	18.15	-6.46	1
TCGA-CJ-5682-01	60	MALE	Stage IV	G4	0	122.83	-1.52	2
TCGA-CJ-5683-01	78	MALE	Stage I	G3	0	62.10	-1.93	2
TCGA-CJ-5684-01	61	MALE	Stage III	G2	0	73.35	1.814	2
TCGA-CJ-5686-01	59	FEMALE	Stage I	G3	0	67.00	7.067	2
TCGA-CJ-5689-01	90	MALE	Stage I	G4	1	53.26	0.536	2
TCGA-CJ-6027-01	77	MALE	Stage I	G4	1	118.85	-4.61	1
TCGA-CJ-6028-01	58	MALE	Stage IV	G4	1	53.42	-0.99	2
TCGA-CJ-6030-01	65	MALE	Stage I	G3	1	75.58	1.242	2
TCGA-CJ-6031-01	54	MALE	Stage I	G3	0	62.66	6.812	2
TCGA-CJ-6032-01	63	FEMALE	Stage II	G3	0	119.64	-1.62	2
TCGA-CJ-6033-01	54	FEMALE	Stage IV	G4	1	7.36	-1.63	2
TCGA-CW-5580-01	73	FEMALE	Stage IV	G3	1	64.57	-2.31	2
TCGA-CW-5581-01	44	MALE	Stage I	G3	0	92.02	-1.73	2
TCGA-CW-5583-01	51	FEMALE	Stage I	G2	0	81.83	-0.06	2
TCGA-CW-5584-01	74	MALE	Stage III	G3	1	5.39	-1.65	2
TCGA-CW-5585-01	51	MALE	Stage IV	G2	0	85.78	-2.52	2
TCGA-CW-5587-01	62	FEMALE	Stage III	G2	0	73.18	0.359	2
TCGA-CW-5588-01	78	FEMALE	Stage I	G2	0	66.31	2.332	2
TCGA-CW-5589-01	52	MALE	Stage I	G2	0	78.18	2.779	2
TCGA-CW-5590-01	51	MALE	Stage IV	G3	1	35.34	-1.78	2
TCGA-CW-5591-01	56	MALE	Stage IV	G2	0	74.66	8.394	2
TCGA-CW-6087-01	61	MALE	Stage IV	G4	1	1.35	3.286	2
TCGA-CW-6088-01	60	MALE	Stage I	G2	0	105.93	-1.19	2
TCGA-CW-6090-01	68	MALE	Stage I	G3	0	83.90	-3.4	2
TCGA-CW-6093-01	73	MALE	Stage I	G1	0	103.43	-0.42	2
TCGA-CW-6097-01	32	MALE	Stage III	G4	1	18.77	1.247	2
TCGA-CZ-4853-01	82	MALE	Stage I	G2	0	25.45	-2.96	2
TCGA-CZ-4854-01	68	MALE	Stage I	G2	1	46.16	-1.45	2
TCGA-CZ-4856-01	62	FEMALE	Stage I	G2	0	0.59	-0.45	2

TCGA-CZ-4857-01	56	MALE	Stage IV	G3	1	47.08	-2.34	2
TCGA-CZ-4858-01	39	MALE	Stage II	G4	1	69.21	-3.55	2
TCGA-CZ-4859-01	59	FEMALE	Stage I	G2	0	58.75	-2.1	2
TCGA-CZ-4860-01	60	MALE	Stage IV	G4	1	6.77	-4.1	2
TCGA-CZ-4861-01	63	MALE	Stage II	G2	1	14.66	-2.78	2
TCGA-CZ-4862-01	46	MALE	Stage I	G2	0	107.54	4.291	2
TCGA-CZ-4863-01	51	FEMALE	Stage III	G3	0	63.39	-1.49	2
TCGA-CZ-4864-01	86	MALE	Stage II	G3	1	93.04	-0.97	2
TCGA-CZ-4865-01	70	FEMALE	Stage I	G2	1	5.46	7.853	2
TCGA-CZ-4866-01	79	FEMALE	Stage I	G3	0	107.41	4.875	2
TCGA-CZ-5451-01	74	MALE	Stage II	G3	0	63.42	-1.16	2
TCGA-CZ-5452-01	69	MALE	Stage II	G2	0	58.82	-1.15	2
TCGA-CZ-5453-01	67	MALE	Stage II	G2	1	79.53	-1.75	2
TCGA-CZ-5454-01	63	MALE	Stage IV	G2	1	23.74	-3.44	2
TCGA-CZ-5455-01	63	MALE	Stage IV	G4	1	18.44	-1.58	2
TCGA-CZ-5456-01	57	MALE	Stage II	G3	0	79.63	-0.53	2
TCGA-CZ-5457-01	62	MALE	Stage III	G4	0	90.54	-0.56	2
TCGA-CZ-5458-01	43	MALE	Stage III	G3	0	91.69	7.584	2
TCGA-CZ-5459-01	63	MALE	Stage III	G3	0	55.33	-0.98	2
TCGA-CZ-5460-01	55	MALE	Stage IV	G2	0	94.45	-1.29	2
TCGA-CZ-5461-01	52	MALE	Stage IV	G4	1	10.85	-2.42	2
TCGA-CZ-5462-01	83	MALE	Stage IV	G3	1	10.22	3.454	2
TCGA-CZ-5463-01	76	MALE	Stage II	G2	0	21.76	-4.36	2
TCGA-CZ-5464-01	69	MALE	Stage IV	G2	0	69.96	5.557	2
TCGA-CZ-5465-01	76	FEMALE	Stage III	G2	1	84.30	-2.19	2
TCGA-CZ-5466-01	67	MALE	Stage III	G2	0	22.52	0.669	2
TCGA-CZ-5467-01	86	FEMALE	Stage III	G4	1	2.40	4.63	2
TCGA-CZ-5468-01	84	MALE	Stage IV	G4	1	1.94	-4.82	1
TCGA-CZ-5469-01	41	MALE	Stage II	G2	1	31.10	-1.28	2
TCGA-CZ-5470-01	72	FEMALE	Stage II	G3	0	12.69	-1.66	2
TCGA-CZ-5982-01	59	FEMALE	Stage I	G2	0	80.19	-2.3	2
TCGA-CZ-5984-01	51	MALE	Stage I	G3	0	67.96	-1.33	2
TCGA-CZ-5985-01	58	MALE	Stage II	G2	0	65.65	6.095	2
TCGA-CZ-5986-01	61	MALE	Stage I	G3	0	12.26	-2.37	2
TCGA-CZ-5987-01	60	MALE	Stage IV	G2	1	14.63	-2.9	2
TCGA-CZ-5988-01	38	MALE	Stage I	G2	0	22.78	-1.6	2
TCGA-CZ-5989-01	60	MALE	Stage II	G2	0	62.63	-3.46	2
TCGA-DV-5565-01	59	MALE	Stage I	G2	0	43.69	1.412	2
TCGA-DV-5566-01	67	FEMALE	Stage I	G2	0	45.96	0.406	2
TCGA-DV-5567-01	40	FEMALE	Stage I	G2	0	65.88	-0.43	2
TCGA-DV-5568-01	26	MALE	Stage I	G2	0	12.16	0.19	2
TCGA-DV-5569-01	29	FEMALE	Stage I	G2	0	11.67	3.271	2
TCGA-DV-5573-01	41	MALE	Stage I	G2	0	37.15	1.746	2
TCGA-DV-5574-01	37	MALE	Stage I	G2	0	66.28	6.005	2
TCGA-DV-5575-01	52	FEMALE	Stage I	G2	0	33.07	-0.59	2
TCGA-DV-5576-01	55	FEMALE	Stage I	G2	1	23.90	4.219	2

TCGA-DV-A4VX-01	59	MALE	Stage IV	G4	1	53.46	-4.6	1
TCGA-DV-A4VZ-01	53	MALE	Stage I	G2	0	12.00	5.437	2
TCGA-DV-A4W0-01	55	MALE	Stage I	G3	0	81.21	-3.25	2
TCGA-DV-A4W0-05	55	MALE	Stage I	G3	0	81.21	-0.57	2
TCGA-EU-5904-01	47	FEMALE	Stage I	G1	0	18.12	-1.07	2
TCGA-EU-5905-01	67	FEMALE	Stage I	G3	0	3.91	0.065	2
TCGA-EU-5906-01	55	MALE	Stage I	G2	0	6.77	-0.43	2
TCGA-EU-5907-01	81	MALE	Stage III	G3	0	4.18	2.629	2
TCGA-G6-A5PC-01	54	FEMALE	Stage IV	G4	1	7.96	-0.83	2
TCGA-G6-A8L6-01	55	MALE	Stage IV	G3	1	10.29	-2.09	2
TCGA-G6-A8L7-01	81	FEMALE	Stage I	G3	0	70.13	-2.86	2
TCGA-G6-A8L8-01	62	FEMALE	Stage I	G3	1	35.87	-5.74	1
TCGA-MM-A564-01	68	MALE	Stage II	G2	0	19.96	-1.96	2
TCGA-MM-A84U-01	58	FEMALE	Stage I	G2	0	23.01	4.618	2
TCGA-MW-A4EC-01	72	FEMALE	Stage I	G2	0	16.37	0.182	2
TCGA-T7-A92I-01	47	FEMALE	Stage I	G1	0	11.70	-4.49	1
